# Development and validation of a nomogram for predicting prostate cancer based on combining contrast-enhanced transrectal ultrasound and biparametric MRI imaging

**DOI:** 10.3389/fonc.2023.1275773

**Published:** 2023-11-17

**Authors:** Wanxian Nong, Qun Huang, Yong Gao

**Affiliations:** Department of Ultrasound, First Affiliated Hospital of Guangxi Medical University, Nanning, Guangxi, China

**Keywords:** prostate cancer, multiparametric MRI, biparametric MRI, contrast-enhanced transrectal ultrasound, PI-RADS

## Abstract

**Objectives:**

This study was to explore the feasibility of combining contrast-enhanced transrectal ultrasound (CE-TRUS) with biparametric MRI (CEUS-BpMRI) score for diagnosing prostate cancer (PCa).

**Methods:**

A total of 183 patients with suspected PCa who underwent multiparametric MRI (Mp-MRI) and CE-TRUS were included. CEUS-BpMRI score was developed based on the results of Mp-MRI and CE-TRUS. The diagnostic performance was evaluated by the area under the curve (AUC). The diagnostic efficacy of the CEUS-BpMRI score, BpMRI score, and PI-RADS v2.1 score were compared. Total patients were randomly assigned to a training cohort (70%) or validation cohort (30%). A nomogram was constructed based on univariate and multivariate logistic regression. The model was evaluated by AUC and calibration curve.

**Results:**

The diagnostic performance of CEUS-BpMRI score (AUC 0.857) was comparable to that of PI-RADS v2.1 (AUC 0.862) (*P* = 0.499), and both were superior to Bp-MRI score (AUC 0.831, *P* < 0.05). In peripheral zone lesions with Bp-MRI score of 3, there was no statistically significant difference between PI-RADS v2.1 score (AUC 0.728) and CEUS-BpMRI score (AUC 0.668) (*P* = 0.479). Multivariate analysis showed that age, total prostate specific antigen/free prostate specific antigen (F/T), time to peak (TTP), and CEUS-BpMRI score were independent factors. The AUC of the nomogram was 0.909 in the training cohort and 0.914 in the validation cohort.

**Conclusions:**

CEUS-BpMRI score has high diagnostic efficacy for diagnosing PCa. A nomogram model established by combining age, F/T, TTP, and CEUS-BpMRI score can achieve the best predictive accuracy for PCa.

## Introduction

Prostate cancer (PCa) is one of the major malignant tumors affecting human health (1). It is the second most common malignant tumor in the world, and the fifth cause of cancer death in men (2). Multiparametric magnetic resonance imaging (Mp-MRI) is currently recognized as the preferred imaging method for the diagnosis of PCa (3). The Prostate Imaging Reporting and Data System (PI-RADS) based on Mp-MRI is a standardized and patterned scoring system for the diagnosis of PCa that is widely used in the risk assessment of PCa (4).

The PI-RADS v2.1 scoring system, updated in 2019, recommends the use of Mp-MRI as the primary diagnostic tool for PCa (5), with T2-weighted imaging (T2WI) and diffusion weighted imaging (DWI) serving as the main sequences, and dynamic contrast-enhanced (DCE) imaging as the supplementary sequence (6). The disadvantages of DCE imaging include prolonged scan time, increased cost, and the risk of potential adverse events such as renal insufficiency or contrast agent allergy (7). Moreover, some studies have shown that Mp-MRI and biparametric magnetic resonance imaging (Bp-MRI) have similar diagnostic efficacy in detecting clinically significant PCa (8). However, other studies have shown that the DCE imaging sequence assists in identifying lesions ≥PI-RADS 3, which include clinically significant PCa, and improves the sensitivity of the PI-RADS scoring system for diagnosing PCa (9). It may be advantageous in achieving more precise risk stratification for PCa (10).

Therefore, there is still a need for a safe and easy test, especially for patients with renal insufficiency or a gadolinium-based contrast agent allergy, as a complementary protocol to Bp-MRI for the diagnosis of PCa. Contrast-enhanced ultrasound (CEUS) is easy to operate and poses no threat to renal function. It is also highly safe, with allergic reactions rarely reported (11). Recent studies have demonstrated that CE-TRUS can effectively enhance the detection of PCa by displaying the microvascular perfusion of the tumor (12). Moreover, the quantitative parameters of CEUS, particularly the time to peak (TTP) and peak intensity (PI), have shown high diagnostic value for PCa (13, 14).

To our knowledge, few studies have shown the use of combining contrast-enhanced transrectal ultrasound (CE-TRUS) with Bp-MRI to construct an imaging scoring system for PCa (15). The aim of this study was to investigate the feasibility of CE-TRUS replacing the DCE imaging sequence and assisting Bp-MRI in the diagnosis of PCa. A nomogram model combining quantitative parameters of CE-TRUS and the CEUS-BpMRI score was established to predict PCa.

## Materials and methods

### Patients

**Figure 1:**
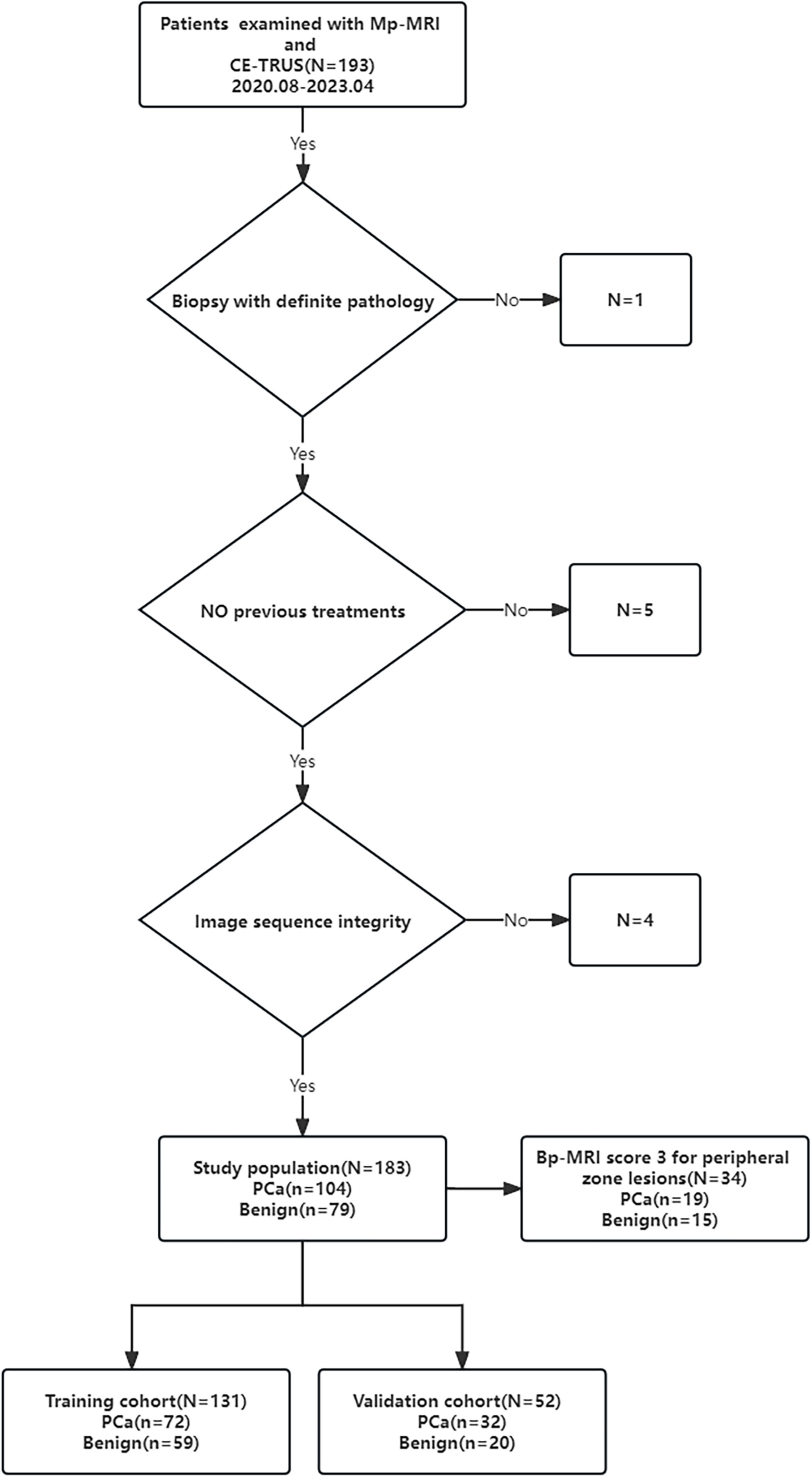
Flowchart shows study inclusion and exclusion criteria.

The study was approved by the Ethics Committee of the hospital (Approval Number 2023-E129-01). The study retrospectively analyzed 183 patients with clinical suspicion of PCa who were admitted to the First Affiliated Hospital of Guangxi Medical University from August 2020 to April 2023. The inclusion criteria for this study were as follows: the prostate specific antigen (PSA) level was greater than or equal to 4 ng/ml, patients all underwent Mp-MRI and CE-TRUS examinations within one week before puncture, and patients all underwent prostate MRI/TRUS fusion-targeted puncture combined with systematic puncture. Pathological diagnosis were obtained through targeted puncture or radical prostatectomy. Patients were excluded if they had poor image quality or incomplete imaging sequences, history of prostate surgery or endocrine therapy, or allergies to the contrast media used in imaging. The processes of inclusion and exclusion of study subjects are shown in **Figure 1**.

### Mp-MRI examination

**Figure 2:**
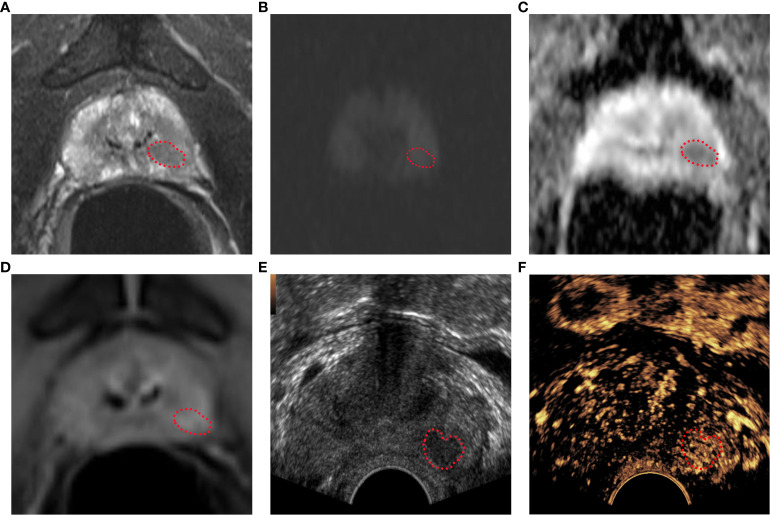
Bp-MRI score, PI-RADS v2.1 score, and CEUS-BpMRI score results display. Bp-MRI score was 3. PI-RADS v2.1 score was 4. The CEUS-BpMRI score was 4. The lesion was marked with a red dashed line. **(A)** T2WI score was 4. **(B)** DWI (B value 1000) and **(C)**ADC. **(D)** DCE: positive. **(E)** Conventional ultrasound. **(F)** CEUS: positive.

MRI examination was performed with a Siemens Viro 3.0T MRI imager and a GE 750 3.0T MRI imager with a body phased coil. The conventional sequences included axial T2WI (field of view 240 mm × 240 mm, slice thickness 4 mm, slice spacing 2.0 mm, TR 4500 ms, TE 85ms), axial T1WI (field of view 240 mm × 24 0mm, slice thickness 4 mm, slice spacing 2.0 mm, TR 700 ms, TR 4500 ms, TE 85 ms), axial T1WI (field of view 240 mm × 240 mm, slice thickness 4 mm, slice spacing 2.0 mm, TR 700 ms, TE 11 ms), axial DWI (b = 0, 1000 s/mm2, TR 5800 ms, TE 86 ms, field of view 240 mm × 240 mm, matrix 192, slice thickness 4.0 mm, slice spacing 2.0 mm, excitation times 3). DCE imaging was performed with a volume interpolated body examination sequence in the axial plane. The scan parameters were TR 5.0 ms, TE 1.7 ms, and flip angle 15°. The field of view was 260 mm × 260 mm, the matrix was 138 × 192, the slice thickness was 2.0 mm, the slice spacing was 0 mm, and the excitation frequency was once. The first phase was equivalent to the T1-mapping sequence. After the injection of the contrast agent, 35 consecutive dynamic contrast-enhanced scans were performed. The total scanning time was about 5 minutes and 30 seconds. The contrast agent was Gd-DTPA (concentration 0.5 mmol/mL, dose 0.2 mmol/kg), injected through the elbow vein with a high-pressure syringe at a flow rate of 3 mL/s. After the contrast injection, the same volume of normal saline was injected. The Mp-MRI imaging results of all subjects were interpreted by two experienced radiologists, and the Bp-MRI score and PI-RADS v2.1 score were determined (**Supplementary Material**, **Figure 2**). When there were differences in the conclusions, a consensus was reached through discussion.

### CE-TRUS examination

The GE Logiq E9 ultrasound diagnostic instrument (GE Healthcare, Milwaukee, WI, USA) was used. The frequency of the transrectal probe IC5-9-D was 3-9 MHz. The CE-TRUS examination was performed by two senior sonographers with 10 years of CEUS experience. On examination, the patient was in the left lateral decubitus position with both legs flexed and his knees folded with his hands. First, a conventional transrectal ultrasound was performed to observe the shape, size, and boundary between the peripheral zone and transitional zone of the prostate and whether there were nodules or abnormal blood flow signals. After real-time fusion of MRI and ultrasound images, the suspicious lesions of the prostate indicated by MRI were located, and CE-TRUS was performed on this plane. The ultrasound contrast agent (Sonovue, Bracco) suspension with a concentration of 2.4 mI was rapidly injected through the median vein of the elbow, followed by rapid flushing with 5 ml of physiological saline. The contrast-enhanced appearance of the prostate was observed for 3 minutes until the contrast agent subsided, then the data were stored in DICOM format. Time Intensity Curve (TIC) curve was drawn, and peak intensity (PI) and time to peak (TTP) were recorded. After outlining the region of interest (ROI), the TIC curve was obtained through the analysis software, as well as the quantitative analysis values of PI and TTP. The CEUS-BpMRI score was determined (**Table 1**, **Figure 2**). The conclusions were reached through discussion when differences were found.

**Table 1:** CEUS-BpMRI score (Peripheral zone).

Bp-MRI score	CEUS	CEUS-BpMRI score
1	Any	1
2		2
3	–	3
	+	3 + 1 = 4
4	Any	4
5		5

### Prostate biopsy and pathology

Prostate biopsy was performed by MRI/TRUS fusion targeted puncture combined with systematic puncture using 18 G needles (CR Bard Inc., Tempe, AZ, USA). Two to three targeted biopsies were performed at the lesion with abnormal MRI indications, combined with 12 systematic needle biopsies. Specimens were marked according to the biopsy site.

### Statistical analysis

SPSS 23.0 software (Armonk, NY: IBM Corp) and R software (Vienna, Austria: R Foundation for Statistical Computing, version 4.1.0, https://www.r-project.org) were used for statistical analysis. The categorical data were analyzed by a chi-square test, and continuous correction was performed when necessary. The continuous data were analyzed by a t test or non-parametric Wilcoxon test according to whether they conformed to the normal distribution. The receiver operating characteristic curve (ROC) was established according to the three scores, and the area under the curve (AUC) was calculated. The diagnostic discrimination was compared and analyzed by the Delong test (*P* < 0.05 was considered statistically significant). The sample of patients were divided into the training cohort and validation cohort at a ration of 7:3. Univariate and multivariate logistic regression analyses were performed to estimate the parameters in the training cohort. The significant variables in the multivariate analysis were included to draw a nomogram to visualize the risk of PCa in patients. The AUC and calibration curve were used to validate the nomogram model in the training cohort and validation cohort.

## Results

### Pathological, clinical, and imaging characteristics of the patients

A total of 183 patients were included in this study, including 104 cases of PCa, 103 cases of prostate adenocarcinoma, 1 case of adenoid basal-cell carcinoma, and 79 cases of benign prostatic diseases, including 64 cases of benign prostatic hyperplasia and 15 cases of benign prostatic hyperplasia with chronic interstitial inflammation.

Among the benign and PCa groups, the age, total prostate specific antigen (T-PSA), free prostate specific antigen (F-PSA), and PI in the PCa group were higher than those in the benign group (*P <* 0.05), and the F/T and TTP of the PCa group were lower than those of the benign group (*P <* 0.05), as shown in **Supplementary Material**. The PI-RADS v2.1 score, Bp-MRI score, and CEUS-BpMRI score were statistically different between the benign and PCa groups (*P <* 0.05), as shown in **Table 2**. There were 34 cases with a Bp-MRI score of 3 for peripheral zone lesions (19 cases of PCa and 15 cases of benign prostatic lesions). The PI-RADS v2.1 score was significantly different between the benign and PCa groups (*P <* 0.05). The CEUS-BpMRI score was not statistically significantly different between the benign and PCa groups (*P* = 0.080), as shown in **Supplementary Material**.

**Table 2:** The PI-RADS v2.1, Bp-MRI score and CEUS-BpMRI score of the patients.

	PCa (n=104)	Benign prostatic lesions (n=79)	P-value
PI-RADS v2.1			
1-2	2	25	<0.001
3	7	30	
4	29	16	
5	66	8	
Bp-MRI score			
1-2	2	25	<0.001
3	22	35	
4	14	11	
5	66	8	
CEUS-BpMRI score			
1-2	2	25	<0.001
3	8	29	
4	28	17	
5	66	8	

### Comparison of the diagnostic efficacy of the scores

**Figure 3:**
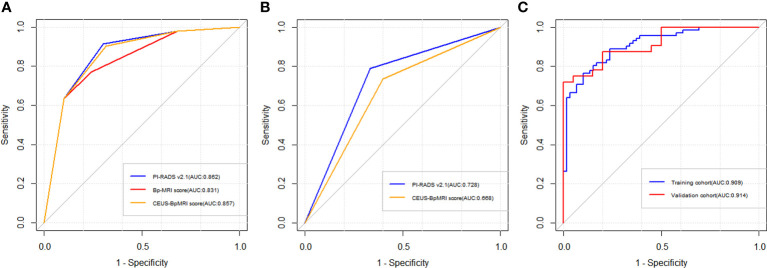
Receiver operating characteristic curve (ROC) curve of three scores. **(A)** The diagnostic efficacy of Bp-MRI score, PI-RADS v2.1 score, and CEUS-BpMRI score in the population. **(B)** The diagnostic efficacy of PI-RADS v2.1 and CEUS-BpMRI scores (Bp-MRI score 3 for peripheral zone lesions). **(C)** The diagnostic efficacy of the nomogram model in the training cohort and validation cohort.

The diagnostic efficacy of the CEUS-BpMRI score (AUC 0.857) was comparable to that of the PI-RADS v2.1 (AUC 0.862) (*P* = 0.499), and both were superior to the Bp-MRI score (AUC 0.831, *P* < 0.05) (**Table 3**, **Figure 3A**). In peripheral zone lesions with a Bp-MRI score of 3, the diagnostic efficacy of the PI-RADS v2.1 score (AUC 0.728) was similar to that of the CEUS-BpMRI score (AUC 0.668) (*P* = 0.479) (**Supplementary Material**, **Figure 3B**).

**Table 3:** Diagnostic efficacy of the three scores.

Three scores	AUC	95%CI	sensitivity	specificity	P-value(Delong test)
①PI-RADS v2.1	0.862	0.809-0.915	0.913	0.696	①vs②: 0.004
②Bp-MRI score	0.831	0.774-0.888	0.635	0.899	②vs③: 0.014
③CEUS-BpMRI score	0.857	0.803-0.911	0.904	0.684	③vs①: 0.499

### Establishing the nomogram model

**Figure 4:**
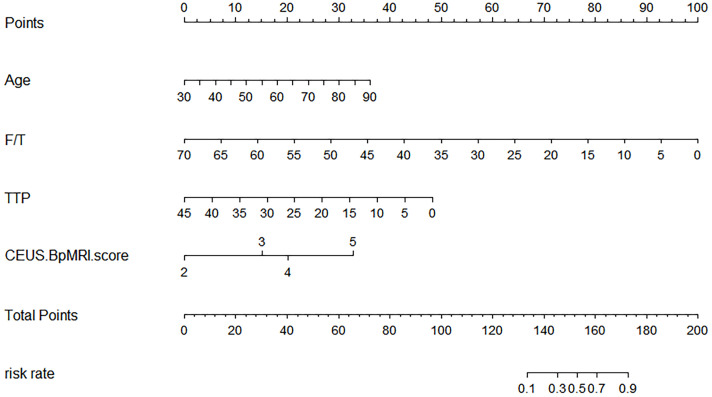
The Nomogram shows a logical model for predicting PCa based on (age, F/T, TTP, and CEUS-BpMRI score).

**Figure 5:**
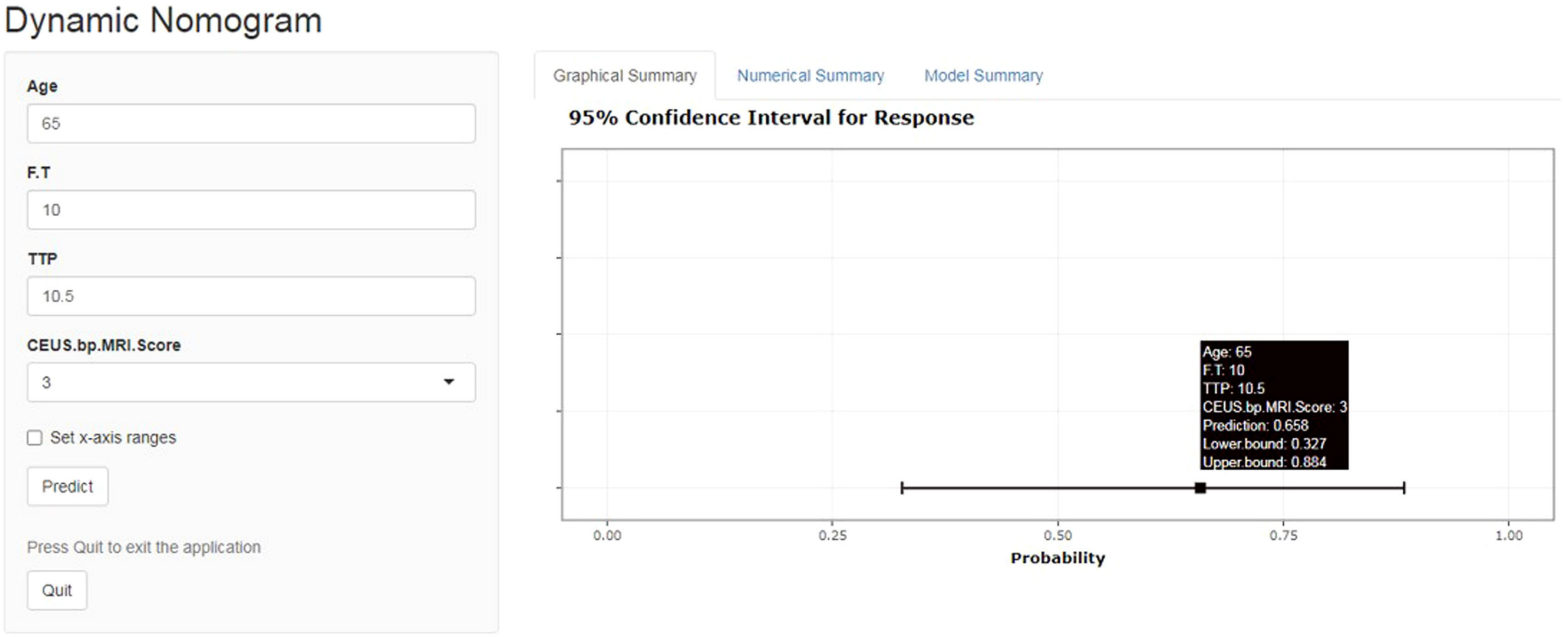
Network dynamic graph based on the Nomogram model (website: https://qwe6524.shinyapps.io/dynnomapp/). The patient was 65 years old and had an F/T of 10%, a contrast-enhanced ultrasound TTP of 10.5 seconds, and a CEUS-BpMRI score of 3 points. The dynamic Normogram estimated the patient’s risk of PCa at 0.658.

**Figure 6:**
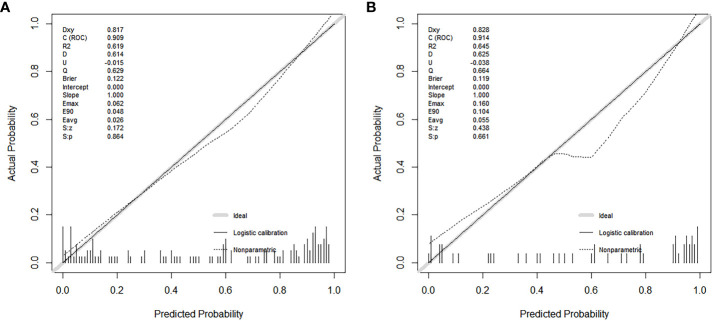
Calibration curves show the model’s effectiveness in predicting the performance of PCa in both the training cohort **(A)** and the validation cohort **(B)**.

A total of 183 patients were divided into a training cohort (n = 131) and validation cohort (n = 52) according to the ratio of 7∶3. There was good agreement between the training and validation cohorts (**Supplementary Material**). Univariate and multivariate logistic regression analyses were performed on the training cohort. Multivariate analysis showed that age, F/T, TTP, and CEUS-BpMRI score were independently associated with PCa, as shown in **Table 4**. Combining these factors, a nomogram (**Figure 4**) and a network dynamic nomogram (**Figure 5**) were created. These showed good discrimination performance in the training cohort and validation cohort, with AUC values of 0.909 and 0.914, respectively (**Table 5**, **Figure 3C**). The calibration curves for the training and validation cohorts are shown in **Figure 6**.

**Table 4:** Univariate and multivariate Logistic regression analysis of the training cohort.

characteristics	Univariate Logistic regression analysis			Multivariate Logistic regression analysis		
	OR	95%CI	P-value	OR	95%CI	P-value
Age	1.048	1.006-1.093	0.026	1.085	1.005-1.171	0.037
T-PSA	1.076	1.033-1.120	<0.001	1.016	0.911-1.134	0.773
F-PSA	1.135	1.030-1.250	0.011	1.306	0.707-2.409	0.394
F/T	0.865	0.810-0.924	<0.001	0.796	0.679-0.932	0.005
PI	1.176	1.081-1.279	<0.001	0.976	0.849-1.122	0.734
TTP	0.882	0.819-0.949	0.001	0.859	0.764-0.967	0.012
CEUS-BpMRI score						
1-2	NA			NA		
3	1.932	0.333-11.203	0.463	12.826	1.101-149.345	0.042
4	13.731	2.717-69.398	0.002	14.891	1.763-125.778	0.013
5	53.429	10.076-283.302	<0.001	33.080	3.423-319.724	0.003

**Table 5:** The diagnostic performance of the model in the training cohort and validation cohort.

Model	AUC	95%CI	sensitivity	specificity	P-value(Delong test)
①training cohort	0.909	0.860-0.957	0.764	0.898	①vs② : 0.904
②validation cohort	0.914	0.842-0.987	0.719	1.000	

## Discussion

Among Mp-MRI sequences, the DCE imaging sequence has a long scanning time and high cost, and it may have the risk of adverse events, such as renal function damage or contrast agent allergy (16). Moreover, some studies have found that Mp-MRI and Bp-MRI have the same diagnostic efficacy for clinically significant PCa (17, 18). In this case, some researchers have shown that Bp-MRI can be used instead of Mp-MRI (19). However, a DCE imaging sequence can help to identify PI-RADS v2.1 ≥ 3 lesions in the peripheral zone, which is beneficial to the risk stratification of PCa (20). Therefore, the examination protocol of CE-TRUS combined with Bp-MRI was used in this study. CEUS is easy to operate, poses no damage to renal function, and has high safety, and allergic reactions are rare (21). The established CEUS-BpMRI score had similar diagnostic efficacy to PI-RADS v2.1 (*P* = 0.499), as well as similar diagnostic efficacy for peripheral zone lesions with Bp-MRI score 3 (*P* = 0.479). Compared with Mp-MRI, this protocol does not require the use of a gadolinium contrast agent and has the advantages of no damage to renal function, high safety, and fewer allergic reactions (22). The CEUS-BpMRI score can achieve similar diagnostic performance to Mp-MRI with fewer side effects.

CEUS has similar diagnostic efficacy to the DCE imaging sequence, which may be related to the contrast medium used. The ultrasound contrast agent (Sonovue) is a microbubble contrast agent (23). PCa tissue has more neovascularization and richer blood flow (24). Sonovue can improve the detection of PCa by showing the microvascular perfusion of PCa (25). Compared with normal prostate tissue, PCa will show early, rapid, and high enhancement on CEUS (26). The gadolinium contrast agent used in DCE imaging sequences is a molecular contrast agent (27). Because PCa tissues usually have higher vascular density and permeability, the gadolinium contrast agent can be absorbed and accumulated in the blood vessels and spaces around the tumor, resulting in significantly high signal intensity in the tumor area and low signal intensity in the surrounding normal tissues (13, 28). Both Sonovue and gadolinium contrast imaging were associated with more abundant neovascularization in PCa, with some similarity (13). Therefore, especially in patients with renal insufficiency or allergy to gadolinium contrast agents, CEUS may be an alternative to DCE imaging sequences.

This study also developed a nomogram based on the CEUS-BpMRI score combined with clinical and CEUS features, which showed high diagnostic efficiency, sensitivity, and specificity in the training cohort and also achieved good performance in the validation cohort, indicating that the nomogram can provide a certain reference for individualized clinical diagnosis or decision-making of PCa patients. This study showed that age, F/T, time to peak, and CEUS-BpMRI score were independently associated with PCa. TTP is a washout parameter that reflects the degree of vascularization of PCa (26), and studies have shown that patients with higher vascular density have earlier peak time (29). However, in our study, T-PSA and F-PSA were not independent indicators for predicting PCa, because T-PSA and F-PSA have low specificity and are easily affected by factors such as prostatic hyperplasia and inflammation (30, 31).

In this study, the fitting line of the calibration curve was close to the reference line in the training cohort. This indicated that the calibration was good, because the predicted value was close to the measured value. In the validation cohort, the fitting line was lower than the reference line, when the predicted values were between 0.5 and 0.8. It suggested that there may be an underestimated risk of PCa in this region. One reason may be that these predicted values were between 3-4 points of the CEUS-BpMRI score, in which was the maximum deviation of the model. It may be necessary to add more valuable factors to improve the predictive ability of this region in the future.

This study has some limitations. First, this was a single-center retrospective study, which is subject to some selection bias. Second, the sample size was relatively small. A multicenter, large-sample data set will be needed to verify the stability of the model. Third, although our study used senior physicians for image evaluation, the results of PI-RADS v2.1 or CEUS are still affected by subjective factors of different readers. Future studies are needed to investigate the consistency of CEUS-BpMRI score used by different readers.

## Conclusion

This study confirms that CEUS can be an effective complement to Bp-MRI in the diagnosis of PCa, especially in patients with renal insufficiency or allergy to gadolinium contrast agents. The combination of CE-TRUS and Bp-MRI has high diagnostic efficacy in the diagnosis of PCa. A nomogram model established by combining age, F/T, TTP, and CEUS-BpMRI scores can achieve the best predictive accuracy for PCa, which can accurately estimate the risk of PCa in patients.

## Data availability statement

The original contributions presented in the study are included in the article/**Supplementary Material**. Further inquiries can be directed to the corresponding author.

## Ethics statement

The studies involving humans were approved by First Affiliated Hospital of Guangxi Medical University. The studies were conducted in accordance with the local legislation and institutional requirements. The ethics committee/institutional review board waived the requirement of written informed consent for participation from the participants or the participants’ legal guardians/next of kin because this study was a retrospective analysis, written informed consent was waved. Written informed consent was not obtained from the individual(s) for the publication of any potentially identifiable images or data included in this article because this study was a retrospective analysis.

## Author contributions

WN: Writing – original draft. QH: Data curation. YG: Funding acquisition, Writing – review & editing.
