# Supplementary material for: Development and validation of a nomogram for predicting prostate cancer based on combining contrast-enhanced transrectal ultrasound and biparametric MRI imaging
Source: Front Oncol. 2023 Nov 17;13:1275773. doi: 10.3389/fonc.2023.1275773 (PMC10691548; doi:10.3389/fonc.2023.1275773)
Supplement: Supplementary file 1 [file Table_1.doc]

**Table 1 Bp-MRI score and PI-RADS v2.1 (peripheral zone)**

| DWI | T2WI | Bp-MRI score | DCE | PI-RADS v2.1 |
| --- | --- | --- | --- | --- |
| 1 | Any | 1 | Any | 1 |
| 2 |  | 2 |  | 2 |
| 3 |  | 3 | - | 3 |
|  |  |  | + | 3+1=4 |
| 4 |  | 4 | Any | 4 |
| 5 |  | 5 |  | 5 |

**Table 2 Patient clinical characteristics and CEUS characteristics**

|  | PCa (n=104) | Benign prostatic lesions (n=79) | *P*-value |
| --- | --- | --- | --- |
| Age(year) | 67.5±8.0 | 63.7±9.3 | 0.003 |
| T-PSA(ng/mL) | 22.4;11.7-78.5 | 9.5;7.6-13.9 | <0.001 |
| F-PSA(ng/mL) | 2.7;1.2-8.8 | 1.7;1.1-2.8 | <0.001 |
| F/T(%) | 11.0;7.0-16.0 | 13.0;18.0-25.0 | <0.001 |
| CEUS characteristics |  |  |  |
| PI(dB） | 21.1±4.6 | 16.9±5.0 | <0.001 |
| TTP(s） | 11.6±4.7 | 16.9±7.6 | <0.001 |
| Pathology Gleason score |  |  |  |
| 3+3 | 7(6.7%) | NA |  |
| 3+4 | 16(15.4%) | NA |  |
| 4+3 | 29(27.9%) | NA |  |
| 4+4 | 28(26.9%) | NA |  |
| 4+5 | 9(8.7%) | NA |  |
| 5+3 | 3(2.9%) | NA |  |
| 5+4 | 7(6.7%) | NA |  |
| 5+5 | 4(3.8%) | NA |  |
| Not applicable | 1(1.0%) | NA |  |

**Table 3 Patients' PI-RADS v2.1 score and CEUS-BpMRI score (Bp-MRI score 3 for peripheral zone lesions)**

|  | PCa(n=19) | Benign prostatic lesions (n=15) | *P*-value |
| --- | --- | --- | --- |
| PI-RADS v2.1 |  |  |  |
| 3 | 4 | 10 | 0.013 |
| 4 | 15 | 5 |  |
| CEUS-BpMRI score |  |  |  |
| 3 | 5 | 9 | 0.080 |
| 4 | 14 | 6 |  |

| PI-RADS v2.1 and CEUS-BpMRI score | AUC | 95%CI | sensitivity | specificity | *P*-value(Delong test) |
| --- | --- | --- | --- | --- | --- |
| ①PI-RADS v2.1 | 0.728 | 0.573-0.883 | 0.789 | 0.667 | ①vs②： 0.479 |
| ②CEUS-BpMRI score | 0.668 | 0.505-0.832 | 0.737 | 0.600 |  |

**Table 4 The diagnostic efficacy of PI-RADS v2.1 and CEUS-BpMRI score (Bp-MRI score 3 for peripheral zone lesions)**

**Table 5 Patient clinical characteristics and CEUS characteristics in training cohort and validation cohort.**

|  | training cohort（n=131） | validation cohort（n=52） | *P*-value |
| --- | --- | --- | --- |
| Age(year) | 66.1±8.9 | 65.1±8.4 | 0.502 |
| T-PSA(ng/mL) | 13.7;8.9-31.2 | 14.2;8.3-53.7 | 0.129 |
| F-PSA(ng/mL) | 2.2;1.1-4.2 | 2.6;1.2-5.8 | 0.141 |
| F/T(%) | 13.0;9.0-18.0 | 16.0;9.3-20.0 | 0.811 |
| CEUS characteristics |  |  |  |
| PI(dB) | 19.0±5.2 | 19.9±5.4 | 0.309 |
| TTP(s) | 13.8±6.2 | 14.1±7.7 | 0.727 |
| CEUS-BpMRI score |  |  | 0.882 |
| 1-2 | 19 | 8 |  |
| 3 | 27 | 10 |  |
| 4 | 34 | 11 |  |
| 5 | 51 | 23 |  |
| Pathology |  |  | 0.418 |
| Malignant | 72 | 32 |  |
| Benign | 59 | 20 |  |

**Table 6** ROC curve coordinates

| Cut-off value | Sensitivity | Specificity | Youden index |
| --- | --- | --- | --- |
| PI-RADS v2.1 |  |  |  |
| 2.5 | 0.316 | 0.981 | 0.297 |
| 3.5 | 0.696 | 0.913 | 0.609 |
| 4.5 | 0.899 | 0.635 | 0.534 |
| Bp-MRI score |  |  |  |
| 2.5 | 0.316 | 0.981 | 0.297 |
| 3.5 | 0.759 | 0.769 | 0.528 |
| 4.5 | 0.899 | 0.635 | 0.534 |
| CEUS-BpMRI score |  |  |  |
| 2.5 | 0.316 | 0.981 | 0.297 |
| 3.5 | 0.684 | 0.904 | 0.588 |
| 4.5 | 0.899 | 0.635 | 0.534 |
